# Supplementary material for: Combining IL-6 and SARS-CoV-2 RNAaemia-based risk stratification for fatal outcomes of COVID-19
Source: PLoS One. 2021 Aug 11;16(8):e0256022. doi: 10.1371/journal.pone.0256022 (PMC8357172; doi:10.1371/journal.pone.0256022)
Supplement: S1 File — (DOCX) [file pone.0256022.s001.docx]

**Supporting information**

**Supplemental Method**

Bronchoalveolar lavage fluid (BALF) was collected within 72 hours after MV introduction. Plasma was also isolated from blood samples collected at the same time as BAL. BALF samples were obtained from the lingula lobe of left lung. BAL was performed by introducing 150 mL of sterile nonbacteriostatic saline solution into a lung subsegment and aspirating back after each one of the aliquots. The return of the first 20-mL aliquot (bronchial fraction) was discarded. BALF samples were stored at -80℃ until assay.

**S1 Fig. Correlation analysis between interleukin (IL)-6 levels in plasma and its levels in bronchoalveolar lavage fluid (BALF).**

IL-6 levels at the same time point in plasma and BALF from patients with critically ill COVID-19 (n=10) were measured with ELISA kit. Individual data are shown as closed circles.
