# Supplementary figures and images for: Combining IL-6 and SARS-CoV-2 RNAaemia-based risk stratification for fatal outcomes of COVID-19
Source: PLoS One. 2021 Aug 11;16(8):e0256022. doi: 10.1371/journal.pone.0256022 (PMC8357172; doi:10.1371/journal.pone.0256022)

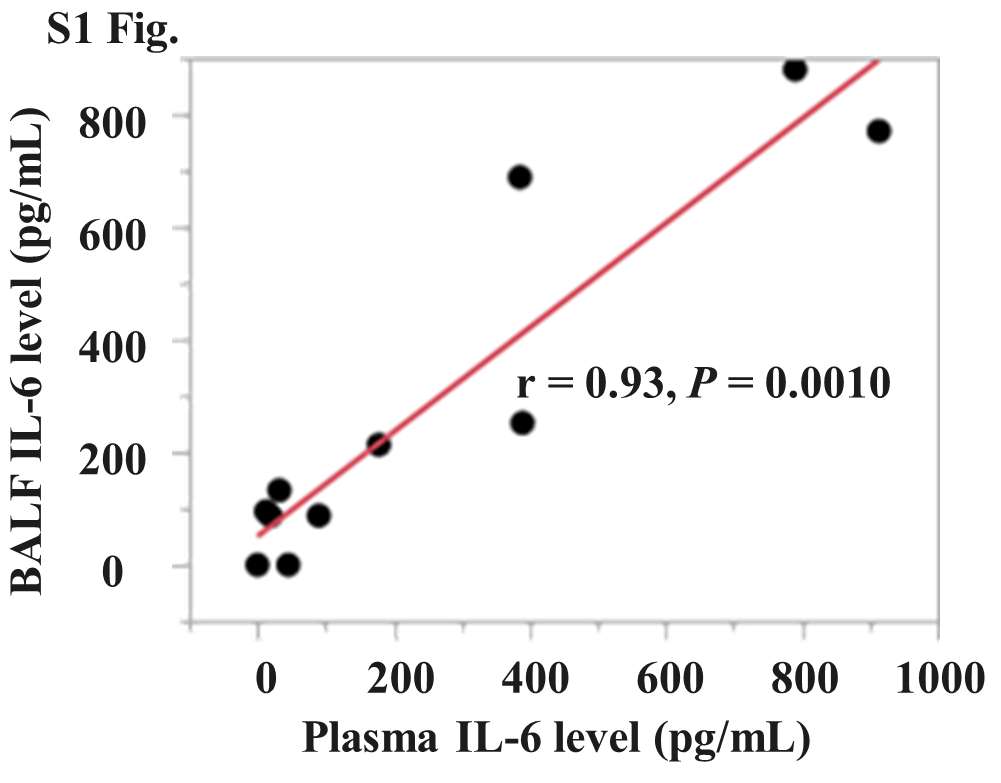

Supplement: S1 Fig — IL-6 levels at the same time point in plasma and BALF from patients with critically ill COVID-19 (n = 10) were measured with ELISA kit. Individual data are shown as closed circles. (TIF) [file pone.0256022.s002.tif]
