# Supplementary material for: Combining IL-6 and SARS-CoV-2 RNAaemia-based risk stratification for fatal outcomes of COVID-19
Source: PLoS One. 2021 Aug 11;16(8):e0256022. doi: 10.1371/journal.pone.0256022 (PMC8357172; doi:10.1371/journal.pone.0256022)
Supplement: Attachment — Submitted filename: Response to Reviewers.docx [file pone.0256022.s003.docx]

**Response to reviewers**

Thank you so much for your great suggestions. We believe that your suggestions extremely improve our manuscript.

According to your suggestions, we revised manuscript as attached new file in this letter.

**(1) Response to reviewer 1**

**Question (Q) #1** Please ensure that your manuscript meets PLOS ONE's style requirements, including those for file naming.

**Answer:** As you suggest, we checked the plos one manual again and made some corrections to the manuscript (Fig. -> Fig). We also made sure that the file name complied with the manual.

Please see result sections of attached new manuscript.

**Q#2-1** Competing Interests

**Answer:** No competing interests exist.

**Q #2-2** Please provide an amended Funding Statement declaring this commercial affiliation, as well as a statement regarding the Role of Funders in your study.

**Answer:** This study was funded by the Japan Agency for Medical Research and Development (JP19fk0108169). This funding organization did not play a role in the study design, data collection and analysis, decision to publish, or preparation of the manuscript and did not provide financial support in the form of authors' salaries. This funding only provided financial support in the form of research materials. This does not alter our adherence to PLOS ONE policies on sharing data and materials.

As you suggest, we updated funding statement and author specific and accurate roles in the Author Contributions section of the online submission form.

**Q #2-3** If your commercial affiliation did play a role in your study, please state and explain this role within your updated Funding Statement.

**Answer:** YM, a contributor is employed by Kanto Chemical Co provided support in the form of salaries, but the Kanto Chemical Co did not have any role in this study, including study design, data collection and analysis, decision to publish, provision of financial support in the form of authors' salaries and research materials, and preparation of the manuscript.

**Q #2-4** Please also provide an updated Competing Interests Statement declaring this commercial affiliation along with any other relevant declarations relating to employment, consultancy, patents, products in development, or marketed products, etc.

**Answer:** Now, we have no competing interests.

**Q#3** Stating the following in the Acknowledgments Section of your manuscript: We will only publish funding information present in the Funding Statement section of the online submission form.

**Answer:** As you suggest, we removed funding information from the Acknowledgments section and included amended statements within cover letter.

Please see the Acknowledgments section in attached new manuscript and cover letter.

**Q#4** Please provide the source and catalog numbers of the PCR assay for SARS-CoV-2 and all ELISA kits used in this study.

**Answer:** As you suggest, we provided the source and catalog numbers of the PCR assay for SARS-CoV-2 and all ELISA kits used in this study into method section.

Please see method section in attached new manuscript as follows;

**(p. 6, line 147)** “ELISA kit (R&D)” to ““ELISA kit (R&D, D6050)”

**(p. 6, line 152)** “QIAamp Viral RNA Mini Kit (QIAGEN)” to “QIAamp Viral RNA Mini Kit (QIAGEN, 52906)”

**(p. 7, line 153)** “N2 primer pairs” to “N2 primer pairs (TaKaRa, XD0008”

**(p. 7, line 157)** “standard product (NIHON GENE RESEARCH LABORATORIES).” To “standard product (NIHON GENE RESEARCH LABORATORIES, JP-NN2-PC).”

**Q#5** Please provide additional details regarding participant consent. In the ethics statement in the Methods and online submission information, please ensure that you have specified:
 - whether consent was obtained
 - whether consent was informed
 - what type of consent you obtained (for instance, written or verbal, and if verbal, how it was documented and witnessed).
 - if your study included minors, state whether you obtained consent from parents or guardians.
 - if the need for consent was waived by the ethics committee, please include this information.”

**Answer:** As you suggested, we newly added informed consent in Ethical considerations.

Please see ethical considerations in the method section (Ethical considerations) of attached new manuscript as follow: **(p. 5, line 106 – line 118)**

“This study was conducted after obtaining ethical clearance from the Institutional Ethics Board of Yokohama City University Hospital (No. B200200048).

During hospitalization, patients were provided negative and positive information regarding this study, including the purpose and contribution of this study, the use of personal information, and complications associated with blood collection, and were asked to participate in this study. Ultimately, we obtained written informed consent for participation in the study and access to medical and laboratory records from patients. Alternatively, we were unable to obtain written consent for participation from four patients who died without being weaned after being placed on a ventilator within hours of admission, which means that we used an opt-out method to obtain consent for this study from them. The study had no risk/negative consequence on those who participated in the study. Medical record numbers were used for data collection and no personal identifiers were collected or used in the research report. Data was accessed from February 9, 2020 to July 28, 2021 and access to the collected information was limited to the principal investigator and confidentiality was maintained throughout the project.”

**Q#6** Please provide the sequences of the N2 primers used in your study.

**Answer:** As you suggest, we provided the sequences of the N2 primers in Materials and Methods section.

Please see the method section of attached new manuscript as follow: **(p.7, line 153 - 157)**

“N2 primer pairs.” to “N2 primer pairs (TaKaRa, XD0008, forward primer: AAATTTTGGGGACCAGGAAC, reverse primer: TGGCAGCTGTGTAGGTCAAC, probe: FAM-ATGTCGCGCATTGGCATGGA-BHQ).”

**Q#7** You state in your manuscript "The researchers also directly communicated with patients or their families to ascertain epidemiological and symptom data." Please state whether a interview guide was used for this purpose. If so, please provide a copy as a supplemental file.

**Answer:** We did not use the interview form. We mentioned this in the method section of detached new manuscript. Please see the Data and Specimen collection of method section as follow: **(p.6, line 139)**

“Interview guide was not used for this purpose.”

**Q#8** In your Methods section, please provide additional information about the participant recruitment method and the demographic details of your participants. Please ensure you have provided sufficient details to replicate the analyses such as:
a) a description of any inclusion/exclusion criteria that were applied to participant recruitment,
b) a statement as to whether your sample can be considered representative of a larger population, and
c) a description of how participants were recruited.

**Answer:** As you suggest, we added descriptions of any inclusion/exclusion criteria, statement of our sample, and the participant recruitment method in the Study setting, design, and population of method section of attached new manuscript as follows:

a) inclusion/exclusion criteria **(p.4, line 95 – line 104)**
“All consecutively admitted patients with the COVID-19 during February 2020 to June 2021 were enrolled in this study. Enrolled patients were observed until 30-day or death after the enrollment to evaluate clinical and laboratory data and clinical outcomes. The primary outcomes were the use of ECMO and in-hospital death after MV use, which were derived from severe ARDS. Patients with missing data, those who died from causes other than ARDS, and those who offered to withdraw from the study were excluded from the evaluation. With complete baseline clinical and laboratory data and outcome data as well as consent for participation, a total of 102 COVID-19 patients were included in the final analysis.”

b) representative of a larger population **(p. 4, line 93 – line 99)**

“This study was a prospective observational study across three hospitals in Japan including Yokohama City University Hospital (YCUH), Yokohama City University Medical Center (YCUMC), and National Hospital Organization Yokohama Medical Center (NHOYMC). All consecutively admitted patients with the COVID-19 during February 2020 to June 2021 were enrolled in this study. The YCUH and YCUMC were mainly intended for severe to critical cases, while NHOYMC was for moderate to severe cases. Therefore, a wide range of cases from moderate to critical were included in the study population.”

c) how participants were recruited **(p. 5, line 108 – line 110)**

“During hospitalization, we provided negative and positive information regarding this study to patients, including purpose and contribution of this study, the use of personal information, and complications associated with blood collection, and asked a participation in this study.”

**Q#9** PLOS requires an ORCID iD.

**Answer:** As you suggest, we have registered ORCID iD of Corresponding Author.

**(2) Response to reviewer 2**

**Q#1**. The authors analyzed data on a cohort of COVID-19 patients admitted in 3 hospitals in Japan in the period February-September 2020, aiming to identify risk factors for a primary composite outcome represented by ECMO and/or 30-days death. Actually, authors dedicated a large part of the paper to describe risk factors associated with MV and in-hospital death, generating a bit confusion for the reader.

**Answer:** As you suggest, we removed mention regarding risk factors associated with mechanical ventilation use from results section **(previous manuscript p.9, line182 – line 199)** and discussion section **(previous manuscript p.17, line303 – line 306)**. Accordingly, Tables 2 and 3 and Figure 1 were also removed, and Tables 4 and 5 in previous manuscript were changed to new Tables 2 and 3, respectively. Figure 2 in previous manuscript was changed to new Figure 1.

Please see new Tables 2 and 3, and new Figure 1 of attached new manuscript.

**Q#2.** Major drawbacks are small sample size: cohort is relatively small (n=83) and, consequently, number of events (n=12) is too limited to support any conclusion.

**Answer 1:** We newly added data from 19 new patients into analysis. However, as you suggest, small sample size is a major limitation of our study. We described study limitation in the discussion and revised overstatement of conclusions.

Please see discussion section of attached new manuscript.

*Study limitation* **(p.17, line 334 - line 336)**

“The present study has several limitations. Further study is needed to validate our results, because our sample size is too small to evaluate optimal cutoffs of variables and determine predictive values of IL-6 and RNAaemia.”

*Overstatement of conclusions* **(p.14, line 276 - line 279)**

We are the first to present this data illustrating that combining IL-6 level, SARS-CoV-2 RNA load, and SpO2/FiO2 ratio in peripheral blood on admission before intubation “is likely to” allow accurate risk stratification of fatal outcomes after the use of MV, including ECMO use and in-hospital death, which is of high relevance for proactive treatment and resource allocation.

*Overstatement of conclusions* **(p.17, line 340 - line 342)**

In conclusion, we are the first to demonstrate that combining IL-6 and viral RNA, together with SpO2/FiO2 ratio, on admission before intubation “is likely to” allows early and accurately predicting fatal outcomes after the use of MV, including ECMO use and in-hospital death.

**Answer 2:** Unfortunately, as IFN-gamma and IL-10 were not measured in additional patients, we inevitably removed data of these cytokines **(previous Fig 1)** and related mention in previous manuscript; method **(previous manuscript p.6, line 132)**, result **(previous manuscript p.9, line 191 -193)** and discussion sections **(previous manuscript p.17, line 303 - line 306)**.

**Q#3.** little novelty: almost all findings about conditions and biomarkers associated with negative COVID-19 outcome have been widely reported in several studies on larger populations and do not add nothing to the current knowledge. Also, the increased risk of negative outcome in patients with detectable SARS-CoV-2 viremia, which remains the most interesting point of the paper, has been already reported

**Answer:** As you suggest, what is new in this study was not defined in previous manuscript. We believe that to our knowledge, biomarkers for accurately and early identifying the risk for ECMO use have yet to be established. Moreover, although High IL-6 and SARS-CoV-2 RNAaemia may reflect pathophysiologic evidence of future deterioration of COVID-19, including hyperinflammation and viral overload, thereby helping us to decide therapeutic strategy, clinical utility of this combination remains uncertain. We added this mention into discussion section.

Please see discussion section of attached new manuscript as follow: **(p.14, line 271 - line 279)**

“High IL-6 and SARS-CoV-2 RNAaemia may reflect pathophysiologic evidence of future deterioration of COVID-19, including hyperinflammation and viral overload, thereby helping us to decide therapeutic strategy. Prognostic utility of combining these biomarkers, however, has not been fully investigated. Particularly, its clinical significance in considering ECMO use was not elucidated. We are the first to present this data illustrating that combining IL-6 level, SARS-CoV-2 RNA load, and SpO2/FiO2 ratio in peripheral blood on admission before intubation is likely to allow accurate risk stratification of fatal outcomes after the use of MV, including ECMO use and in-hospital death, which is of high relevance for proactive treatment and resource allocation.”

**Q#4.** authors declared that criteria for MV and ECMO were PaO2/FiO2 less than 200 and 150, respectively. Really? It seems a quite aggressive approach

**Answer:** In patients with ARDS other than COVID-19, we introduce ventilator or ECMO based on our overall judgment. Therefore, in many cases, even if the P/F ratio is below our standard, we do not introduce a ventilator. However, patients with ARDS due to COVID-19 require more preparations from the viewpoint of infection protection for medical personnel, and it is difficult to introduce them as needed, especially at night when there is less manpower. For this reason, we use the P/F ratio to make decisions on introduction as early as possible.
